# Nephrophobia: a retrospective study of medical students’ attitudes towards nephrology education

**DOI:** 10.1186/s12909-022-03713-z

**Published:** 2022-09-09

**Authors:** William Hull, Emilia Jewell, Shazia Shabir, Richard Borrows

**Affiliations:** 1 Department of Renal Medicine Queen, Elizabeth Hospital Birmingham, Mindelsohn Way, Birmingham, B15 2TH UK; 2 University of Birmingham Medical School, Vincent Drive, Birmingham, B15 2TT UK; 3 North Middlesex University Hospitals, Sterling Way, London, N18 1QX UK

**Keywords:** Education, Nephrophobia, Specialty training, Career, Training

## Abstract

**Background:**

Nephrology is a subject which is challenged by a lack of applicants for trainee places. This study addresses the attitudes of medical students towards the subject and explores the causes of this lack of interest amongst potential specialty trainees.

**Methods:**

Students were asked to complete a survey ranking their attitudes towards nephrology and other specialties. This data was collated and analysed to show trends and allow comparison of the attitudes towards each specialty.

**Results:**

Students felt that along with geriatrics, their least favourite subject was nephrology. Students felt unconfident in diagnosing, managing and understanding chronic conditions more so than acute conditions. Nephrology was consistently amongst the least popular subject for all areas of diagnosis, management and pathophysiology. Renal anatomy was the only area of nephrology that students felt confident in. The less popular specialties of nephrology and geriatrics had the greatest room for improvement when directly instructed in specialty medical training.

**Conclusions:**

Nephrology remains a problematic and unpopular specialty for medical students, driving their unwillingness to consider it as a future career route. This study identifies areas of misconception amongst medical students toward the specialty and highlights areas for improvement in renal training for students.

**Supplementary Information:**

The online version contains supplementary material available at 10.1186/s12909-022-03713-z.

## Background

Nephrology as a specialty has seen a shortage in entry to training across the UK, US, Canada and Australia [1, 2]. There have been a number of attempts to encourage trainees to choose nephrology and establish what may be a driver for this reduction in interest [1, 3, 4] but have to date not come to a definitive conclusion.

‘Neurophobia’ is a term coined by Flanagan et al. [5] to describe the discomfort of medical students and junior doctors when diagnosing and managing neurology patients. The work of Flanagan et al. was followed up with complementary work in the United States by Zinchuk et al. [6] proving this to be a global issue. Both of these works utilised a survey to gauge impression of neurology compared with other specialties, including nephrology. An interesting point to be taken from both of these studies is that nephrology, along with rheumatology, consistently ranked second only to neurology, in terms of discomfort of the respondents [5, 6].

Although questions may arise in regard to the most appropriate method of displaying and interpreting data from such studies [5, 6], these works nevertheless laid strong foundations in both strategy and methodology for similar work in other specialties [7]. However, to date, other studies of students’ opinions of and attitudes towards specialties other than neurology, and certainly in nephrology specifically, are lacking. One study of medical residents conducted in the United States by Nakhoul et al. [8] took a semi-structured interview approach to examining the issues that these residents had surrounding renal medicine. The authors found that factors such as lack of quantity and quality of renal education were indicators for negative opinions of the specialty. Also, there was a negative citation among respondents relating to the patient population, and a perceived lack of ability to ‘make a difference’ to those patients. Nephrology was seen to be highly academic and complex, but such positives seemed not to offset the negative features of the subject.

We propose the term ‘nephrophobia’ to describe the negative attitudes of learners towards nephrology education, training and practice. The current study sought to confirm the limited data that does exist [5, 6, 8], and then extend into relevant domains of clinical competence and confidence, teaching methods, and influences upon future career options [8]. The literature that does exist, examines the opinions of medical trainees post medical school, despite the strong influence of early phases medical education upon later speciality-choice is well recognised [9]. Given this, it seems only logical that the opinions and impressions of medical students should be accounted for, thereby targeting approaches and interventions at an early stage. With established junior doctors negatively citing their educational experiences in nephrology [8] we sought to evaluate the early educational experiences of medical students specifically. We took the opportunity to evaluate the varied components of learning unique to renal medicine, and also extended and expanded upon prior data to include assessment of students’ perceptions of nephrologists themselves, as well as the speciality.

## Methods

A survey, based around that used by Flanagan et al. [5] for neurology but adapted to focus on nephrology was distributed to medical students at the University of Birmingham Medical School (UK). Following conformation that methodology met relevant guidelines and regulations, permission from the University of Birmingham Medical School medical education research committee was granted, requests for interest in completing a survey were circulated via social media and internal email systems to all students studying for MBChB medicine. Individuals responding positively to this approach were then sent the survey itself in a single round of data collection during July 2020. Informed consent was obtained from all participants for participation in the study. Inclusion of anonymised data was indicated by completion of the survey, having been given details of the nature of the survey, no personal identifiable data were collected.

This survey consisted of nine questions relating to their impressions of the field of nephrology and comparing them to other common specialty choices (Appendix 1). The root of the question was a statement e.g. ‘I have a good knowledge of this subject…’ followed by an operator, e.g. one of the specialty areas, cardiology, respiratory, gastroenterology, neurology, rheumatology, endocrinology, geriatrics and nephrology. Next, respondents were given a scale of 5 options from ‘strongly agree’, ‘agree’, ‘neither agree nor disagree’, ‘disagree’ through finally to ‘strongly disagree’ to rate their agreement with the statement in question. For assessment of confidence in diagnosis, management and understanding of pathology, example conditions for both acute and chronic presentations for each specialty were presented as the operators. The next root statement covered comprehension and importance of teaching on specific areas of renal learning for anatomy, physiology, interpreting investigations, dialysis, pharmacology, surgery/transplantation, interactions with other body systems, as adapted from Flanagan et al. [5]. The next area was also adapted from Flanagan et al. [5] to establish students’ feelings on the best ways to learn nephrology. The penultimate question was built using the work of Robbins et al. [10] with influence from Smith et al. [11] and Reed et al. [9] looking at areas students valued in career choices. Finally, the last question was created by the authors to investigate what we believe to be a previously unexplored area: medical students’ perceptions of nephrologists themselves as role models. This data was then coded into numerical data where + 2 was strongly agree and -2 was strongly disagree with 0 representing neither agree nor disagree for the purposes of display, the average score of all respondents is presented as a mean. A final question asked students to specify their gender and their year of study.

At the University of Birmingham, students undertake a module of renal physiology, fluid balance and electrolyte homeostasis in their first year and their clinical rotation on the renal ward (incl. dialysis) is carried out in the 4^th^ year of study. Because of the timing of the questionnaire, at the end of the academic year, all participants in the 1^st^ year and above could be said to have completed their pre-clinical renal module and all 4^th^ and 5^th^ year students (73/121, 60.3%) could be said to have completed their clinical rotation on the renal ward where students shadow junior doctors, attend ward rounds and small group teachings with senior doctors. It is important to say that due to the large number of hospitals served by the University of Birmingham Medical School, it is impossible to encapsulate the experiences of all students, but this does increase the generalisability of this study given the breadth of experience students will have been exposed to. And so although this work represents results from a single academic centre, clinical exposure is represented by 12 hospitals across the West Midlands region of the UK.

Students are identified by their last completed clinical/pre-clinical year, therefore students who were intercalating between 3^rd^ and 4^th^ years were classified as 3^rd^ year students and therefore as having pre-clinical renal exposure only. Graduate entry at the University of Birmingham has one year of pre-clinical followed by integration within the 3^rd^ year undergraduate cohort (totalling 4 years), therefore any graduate entry students in their 1^st^ or 2^nd^ years were classified as having pre-clinical renal exposure only and those in their 3^rd^ and 4^th^ years had completed their clinical renal placements.

Medical students at the University of Birmingham undergo two years (one year for graduate entry students) of pre-clinical teaching in the format of lectures and small group teaching before progressing to complete three years of clinical exposure and specialty medicine and surgery. Pre-clinical renal teaching is mainly taught by clinicians from the Queen Elizabeth Hospital Birmingham and academics from the University of Birmingham Institute of Clinical Sciences and represent both senior clinicians and experienced researchers, all of whom are senior lecturer or above within the university. The University of Birmingham Medical School is one of the largest and most diverse medical schools in the UK with a student body exceeding 2,000 medical students from across the UK and the globe. The city of Birmingham represents a population of 1.15 million with the wider Birmingham conurbation also including Dudley, Solihull, Wolverhampton, Walsall and numerous other population hubs within the West Midlands. The Medical school has students attending clinical teaching with The Wye Valley, Sandwell and West Birmingham, University Hospitals Birmingham, Birmingham Women’s and Children’s, The Dudley group, The Royal Wolverhampton, Walsall Healthcare, Worcester acute hospitals trusts in addition to two mental healthcare trusts (Birmingham and Solihull, and The Black Country) and primary healthcare providers across the West Midlands. Due to the large area covered, students participating in this survey have a wide and varied exposure to clinical nephrology teaching, from tertiary to community settings.

All data was collated with GraphPad™ Prism® 9 statistical software for mac (GraphPad™ Software, San Diego, Ca. USA) and analysed using Chi squared test for trend with type 1 error rate below 5% considered statistically significant (*p* < 0.05). Post-hoc testing of scores relating to nephrology versus other specialities (*n* = 7) was then undertaken, with Bonferoni post-hoc adjustment was used as an alternative analysis, using 7 as the degrees of freedom, and strict statistical significance set at *p* < 0.007. In addition, and to keep consistency with previous work [5, 6] as described above, we also coded data numerically with a range + 2 (‘strongly agree’) through 1, 0, -1 to -2 (‘strongly disagree). For purposes of figurative presentation, and also aligning with previous work for comparison [5, 6] average scores for all respondents is presented as means and standard errors of the mean (SEM).

## Results

Of 165 individuals who expressed an interest in the study, and who were sent the survey, 121 people completed the survey representing a return rate of 73.4%. In response to the question regarding demographics (which were the final questions of the survey thereby minimising any priming effects), 62/121 (51.2%) were female and 48/121 (39.7%) were prior to the medical speciality-specific (including nephrology) module of the course.

### Domain 1: Knowledge

**Fig. 1:**
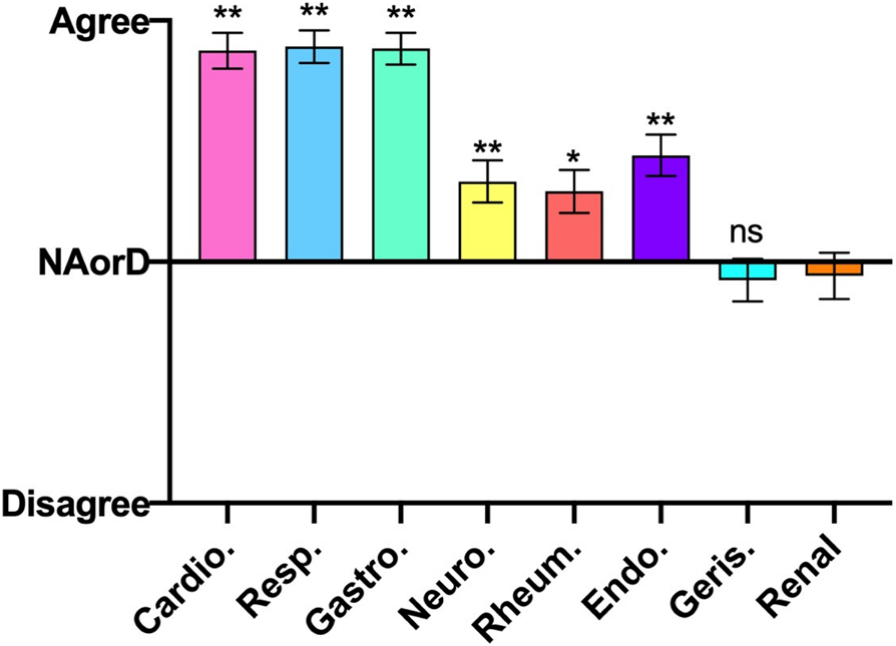
Student responses to the root statement ‘I have a good knowledge of this subject’ for each specialty. NAorD, ‘neither agree nor disagree’. Data is presented as mean score ± SEM. * *P* < 0.05, ** *P* < 0.007 compared to renal

In response to the root statement: ‘I have a good knowledge of this subject’, only two specialties were cited with overall neutral or negative responses: nephrology and geriatrics (Fig. 1). There was no statistical significance between the two negatively cited specialties but compared to the ‘core’ specialties (cardiology, respiratory and gastroenterology), nephrology was highly significantly (*p* < 0.0001) negatively cited and displayed a significant negative citing compared to all other specialties (*p* = 0.0085-*p* = 0.0002) with the exception of geriatrics (*p* = 0.8941).

### Domain 2: Confidence in diagnosis

**Fig. 2:**
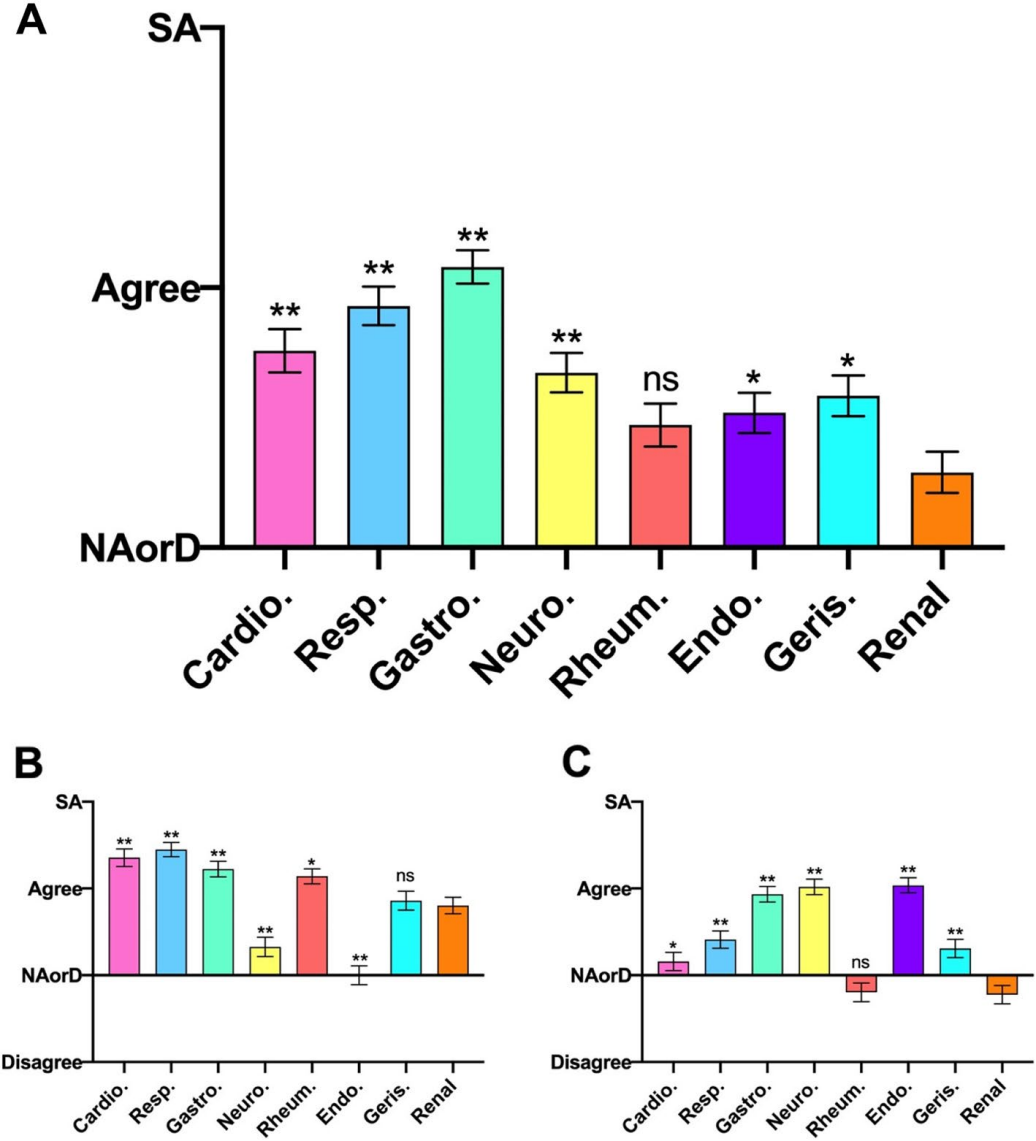
Student responses to the root statement ‘I would be confident in diagnosing a patient with this condition’ for (A – Overall, B – Acute, C – Chronic) presentations in each specialty. SA, Strongly Agree. NAorD, ‘neither agree nor disagree’. Data is presented as mean score ± SEM. * *P* < 0.05, ** *P* < 0.007 compared to renal

In response to the root statement: ‘I would be confident in diagnosing a patient with this condition’, respondents showed a significantly poorer confidence in diagnosing both acute and chronic renal conditions compared with all other specialties with the exception of rheumatology (*p* = 0.1128), as displayed in Fig. 2A.

When respondents’ attitudes to diagnosis of acute conditions was assessed (Fig. 2B), nephrology was rated as the speciality for which students were third-least confident in regard to diagnosis (statistically ‘above’ endocrinology and neurology). All ‘core’ specialties showed a significantly higher rating than nephrology. Both the geriatric and rheumatological presentations showed no statistical significance when compared to nephrology but were numerically higher.

For confidence in diagnosing chronic presentations (Fig. 2C), nephrology ranked least among all the specialties, all other specialties except rheumatology cited significantly more positively in comparison.

#### Domain 3: Confidence in management

**Fig. 3:**
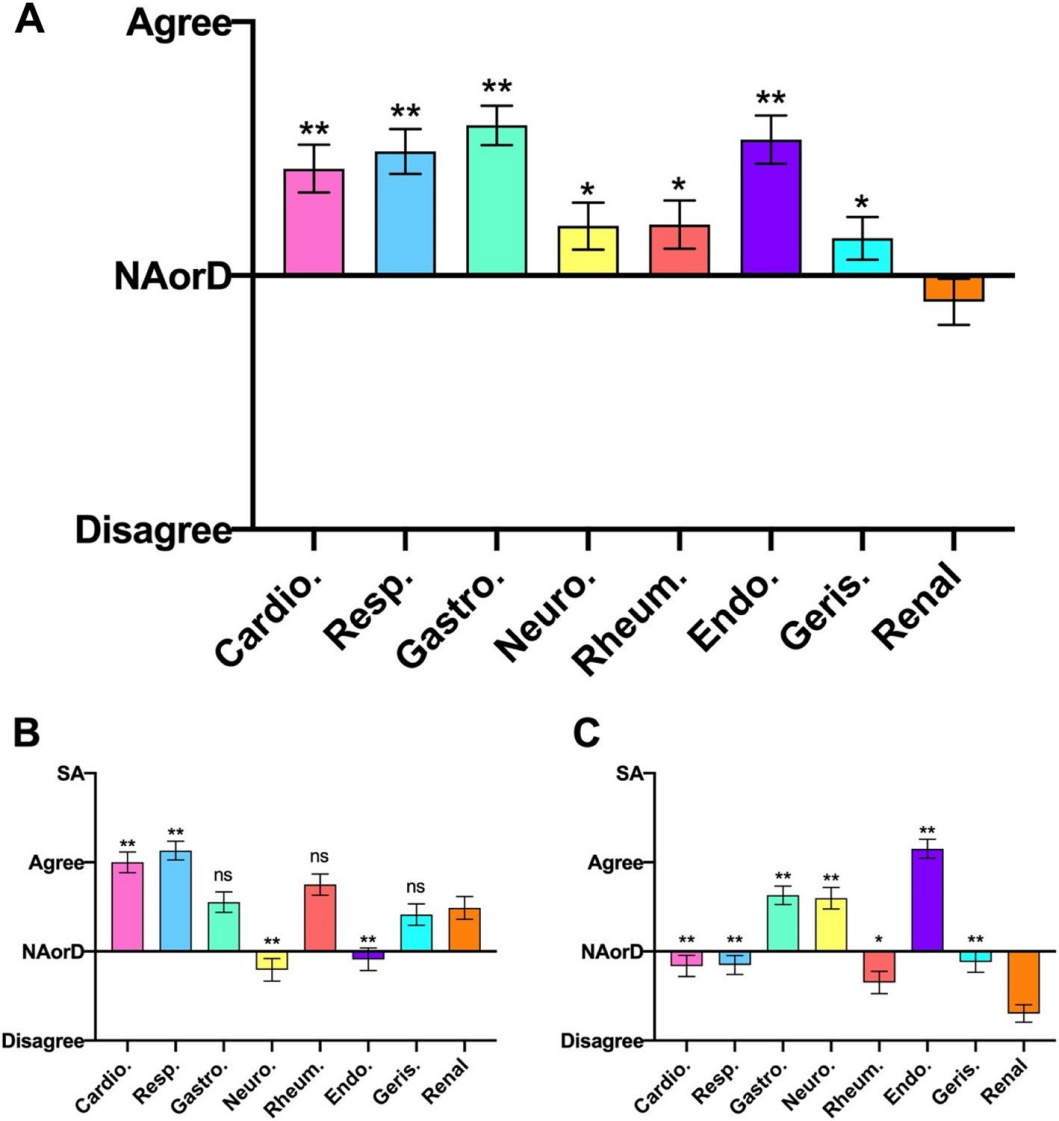
Student responses to the root statement ‘I would be confident in managing a patient with this condition’ for (A – Overall, B – Acute, C – Chronic) presentations in each specialty. SA, Strongly Agree. NAorD, ‘neither agree nor disagree’. Data is presented as mean score ± SEM. * *P* < 0.05, ** *P* < 0.007 compared to renal

In regard to overall confidence in management, nephrology was seen as the only specialty to return an overall neutral or negative response, as shown in Fig. 3A. The responses were significantly more negative in comparison with cardiology, respiratory, gastroenterology and endocrinology.

For management of acute presentations (Fig. 3B) nephrology ranked 4^th^ behind cardiology, respiratory and rheumatology. Both cardiology (*p* = 0.0037) and respiratory (*p* = 0.0002) showed a statistically significantly higher ranking than nephrology.

Confidence in management of chronic conditions (Fig. 3C) was least for nephrology. Four of the other specialties received overall negative ratings (cardiology, respiratory and geriatrics, with rheumatology the only speciality not statistically different to nephrology). Endocrinology, gastroenterology and neurology all received positive ratings, all ranked significantly higher than nephrology.

### Domain 4: Understanding pathophysiology

**Fig. 4:**
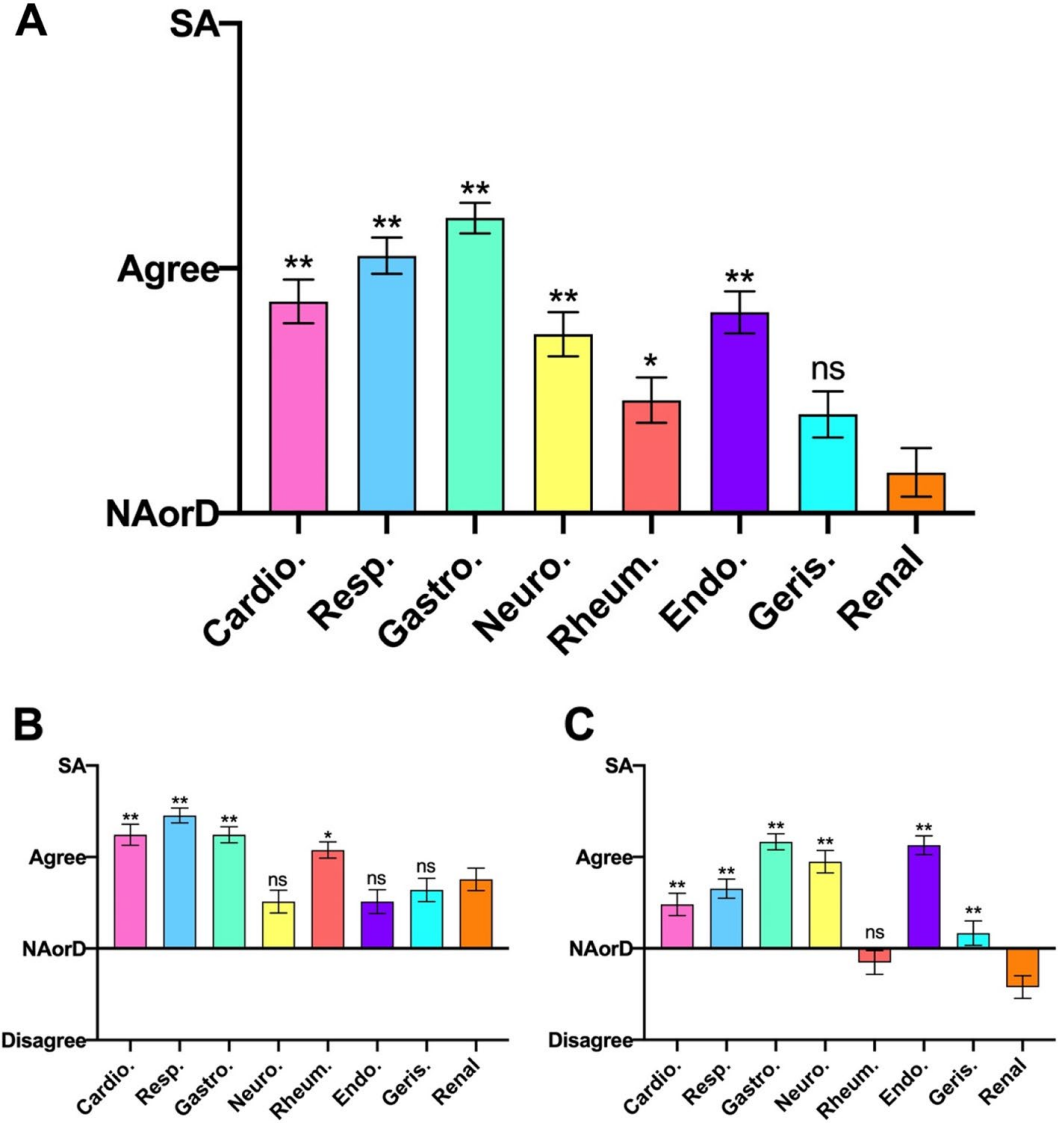
Student responses to the root statement ‘I have a good understanding of the pathophysiology and aetiology of this condition’ for (A – Overall, B – Acute, C – Chronic) presentations in each specialty. SA, Strongly Agree. NAorD, ‘neither agree nor disagree’. Data is presented as mean score ± SEM. * *P* < 0.05, ** *P* < 0.007 compared to renal

Total respondent scores for understanding pathophysiology (Fig. 4A), saw nephrology returned as the lowest ranked specialty. Nephrology knowledge was considered significantly weaker than all specialties other than rheumatology.

In regard to pathophysiology of acute conditions (Fig. 4B) nephrology was in mid position: geriatrics, endocrinology and neurology all ranked below nephrology but not significantly so; respiratory, cardiology and gastroenterology were rated significantly higher than nephrology.

Differences in respondents’ understanding of the pathophysiology of chronic conditions (Fig. 4C) were starker. Nephrology ranked last, and statistically lower than all other specialities with the exception of rheumatology, the only other speciality to return aggregate neutral or negative responses.

### Domains 5: Ease of comprehension

**Fig. 5:**
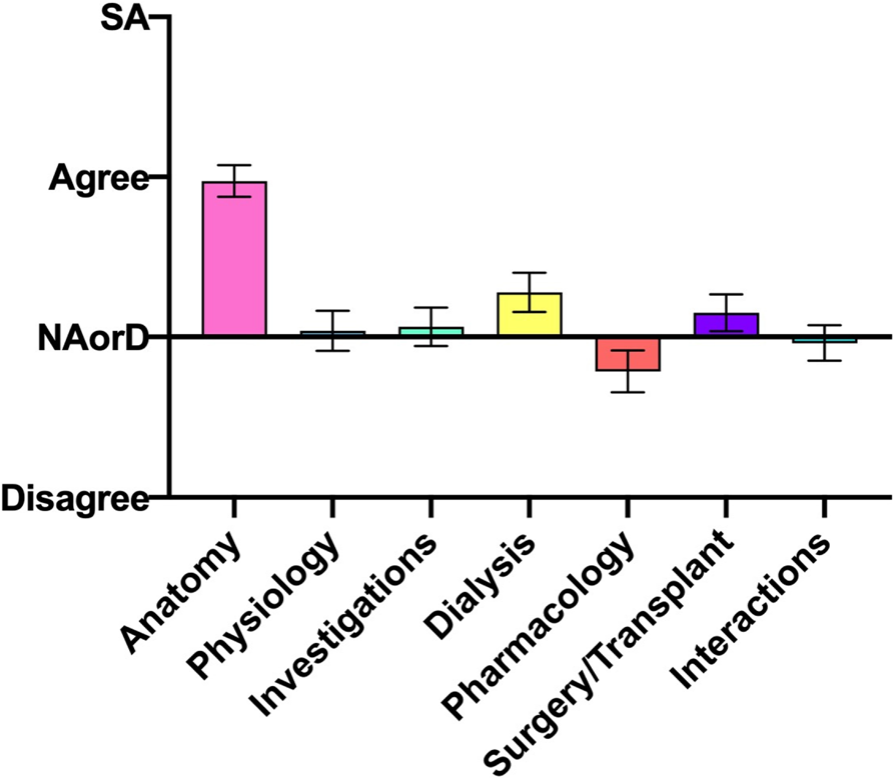
Student responses to the root statement ‘I find this area of nephrology easy to comprehend’. SA, Strongly Agree. NAorD, ‘neither agree nor disagree’. Data is presented as mean score ± SEM

When the different areas of nephrological comprehension were assessed, the only real strength was for renal anatomy (Fig. 5). For other areas, students returned on average somewhat neutral responses, in general neither agreeing nor disagreeing, albeit with some variation.

### Domain 6: Teaching preferences

**Fig. 6:**
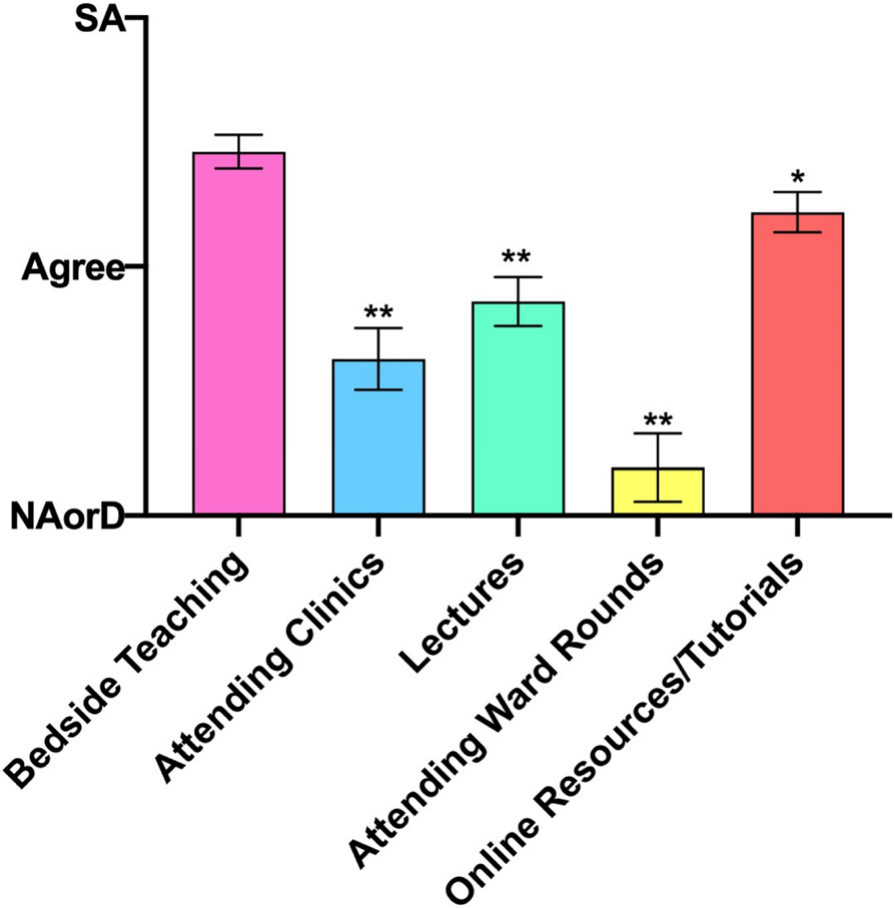
A. Student responses to the root statement ‘This is a good way to learn nephrology’. SA, Strongly Agree. NAorD, ‘neither agree nor disagree’. Data is presented as mean score ± SEM. * *P* < 0.05, ** *P* < 0.001 (Bonferoni adjustment for 4 df) compared to ‘bedside teaching’

In response to the root statement: ‘This is a good was to learn nephrology’, respondents’ preferences for teaching methods suggested attending ward rounds was the least popular and bedside teaching the most popular modality (Fig. 6). With bedside teaching, the most popular, there was a statistical significance to both of the next closest methods, online resources (*p* = 0.0222) and lectures (*p* < 0.0001). However, all methods were rated positively overall, indicating that they do have some utility in nephrological teaching.

### Domain 7: Attractive features of nephrology

**Fig. 7:**
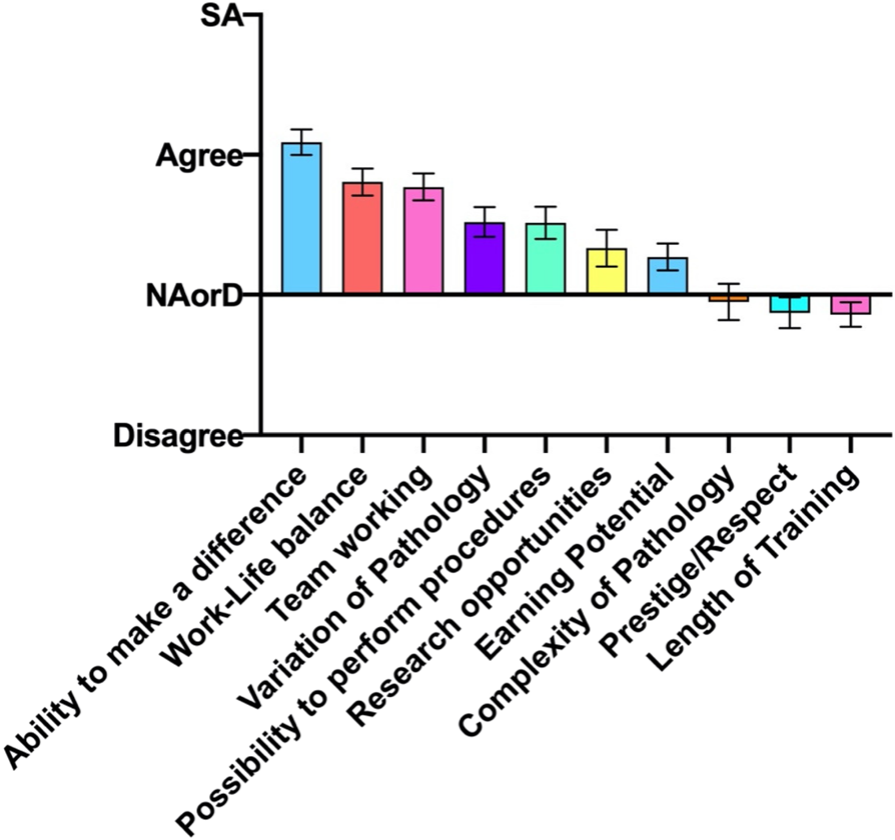
A. Student responses to the root statement ‘This factor of renal practice attracts me to the field’. SA, Strongly Agree. NAorD, ‘neither agree nor disagree’. Data is presented as mean score ± SEM

The factors affecting respondents’ attitudes to nephrology as a career option returned overall negative or neutral scores for length of training prestige/respect and complexity of pathology (Fig. 7). The most positive aspects of a career in nephrology were the ability to make a difference to patients, work-life balance and teamworking.

### Domain 8: Attributes of nephrologists

**Fig. 8:**
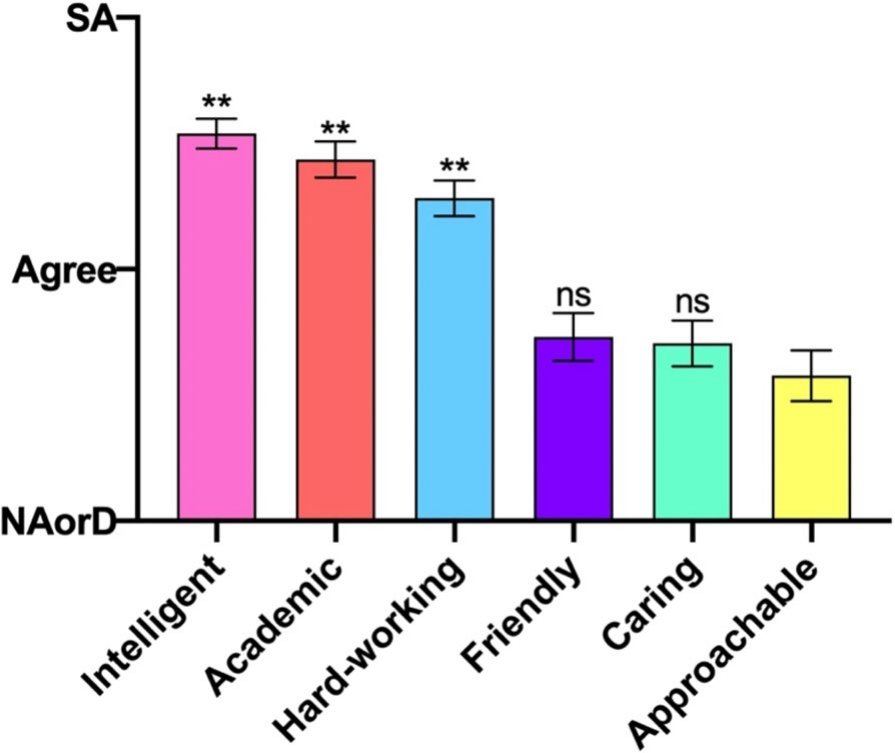
A. Student responses to the root statement ‘When I think of nephrologists, I consider them be…’. SA, Strongly Agree. NAorD, ‘neither agree nor disagree’. Data is presented as mean score ± SEM. * *P* < 0.05, ** *P* < 0.01 (Bonferoni adjustment for 4 df) compared to ‘approachable’

An assessment of respondents’ attitudes towards nephrologists themselves (Fig. 8) showed especially positive responses in regard to being ‘hard-working’, ‘academic’ and ‘intelligent’ (all highly significant against approachability, *p* < 0.0001). Though still cited positively, other domains of ‘friendliness’, ‘caring’ and ‘approachability’ received lower scores in comparison, with no statistical significance against approachability (friendliness, *p* = 0.2638 and caring, *p* = 0.3445).

These results though are all in the ‘positive’ side of the responses meaning that the respondents felt nephrologists to be all of these features, but some more than others.

### Analysis of domain 1: Variation by stage of training

**Fig. 9:**
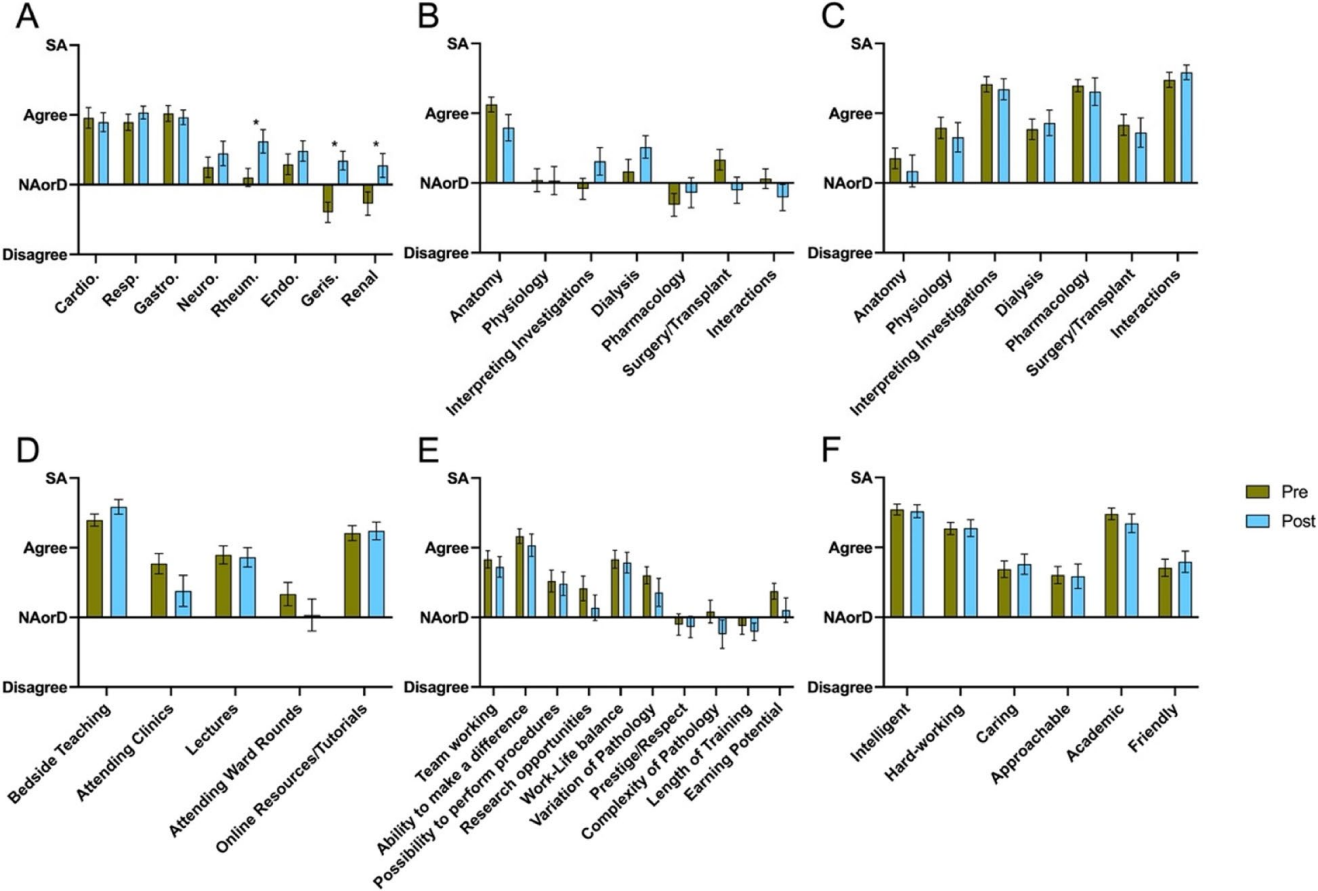
Breakdown analysis of data by pre or post- specialty medicine training. Student responses to the root statements for questions 1, 5, 6, 7, 8 & 9 for each specialty. SA, ‘Strongly Agree’. NAorD, ‘neither agree nor disagree’. Data is presented as mean score ± SEM. * *P* < 0.05

Further breakdown analysis of the date for the root statement ‘I have a good knowledge of this subject’ (survey question 1) differed by stage of training—before or after specialty medicine blocks (Fig. 9A). Students displayed a statistically significant increase in knowledge of nephrology, rheumatology and geriatrics following specialty medicine training comprising these domains. A small but non-significant increase in confidence was noted for neurology and endocrinology, whereas no difference was evident in the cases of cardiology, respiratory and gastroenterology.

Similar analyses (Fig. 9B-F) was carried out for all other survey questions: no significant differences were seen across other domains in regard to either stage of training or self-reported gender.

## Discussion

The future of medicine depends on the next generation of medical students, and the future of individual medical specialities depends on the attitudes of this next generation towards that speciality. The current study extends findings from the field of neurology, where the term ‘neurophobia’ pertains to the fears of learning (and by implication then practicing) neurology [5, 6]. We propose the term ‘nephrophobia’ to describe the equivalent scenario within renal medical training, this time addressing attitudes of UK medical students at a large, diverse, urban university. It addresses not only issues with academic training, but also perspectives on the profession itself, with teaching methods and with nephrologists themselves.

In essence, students report least confidence in their studies of nephrology, where the speciality is well adrift of other core medical specialities including, cardiology, respiratory medicine and gastroenterology. There is no question this is disappointing to the (nephrology) research group who have undertaken this work; we hope that having this survey co-devised by and administered a medical student (WH) without a speciality affiliation, along with transparency of data presentation, we minimised introducing excessive bias in its intepretation.

More specifically, further breakdown and lateral comparison displays that there is a lack of confidence in all areas of management, diagnosis and pathophysiology in nephrology, and especially in the context of chronic (rather than acute) disease, although this bias against chronic disease was evident across all specialities with the exception of endocrinology, something already seen as an area for improvement in all areas of medical teaching [12].

In regard to the areas covered in nephrological teaching, we can see that with the exception of renal anatomy (which is studied during the ‘pre-clinical’ phase) students in general reported neutral responses for domains such as renal physiology, investigations, dialysis, pharmacology, transplantation and interactions with other body systems. Taken together, all these (‘negative’) findings should offer ample opportunity to address curricula and topics within nephrology to target the areas and conditions which seem to hinder students’ education.

The attitudes expressed here lead to a discussion of the optimal ways to deliver nephrological education. Although variability in responses was understandably evident, bedside teaching and online resources were the top two modalities which students found useful. Clearly the former has always been a traditional and effective way to deliver education, albeit logistically more challenging and time consuming for the clinical teacher. Nevertheless, this resonates with the notion that students wish to better understand the clinical context of nephrology as opposed to solely theoretical teaching. These may be more suited to addressing issues in an acute setting, rather than chronic, so this may be a bias generated by the students’ aversion to chronic illnesses as noticed above. Though acute nephrological presentations are important to learn [13], it must not be at the detriment of a holistic approach to renal medicine.

Online tutorials and resources were, of course, in their infancy even 20 years ago, but have been widely adopted by the current generation of ‘heutagogic’ (technologically capable) learners. It may be relevant that this survey was conducted just after the initial wave of COVID-19 infections and subsequent ‘lockdown’ across the UK. As a consequence, much teaching reverted to ‘virtual’ formats. Students’ willingness to adopt these formats may well bode well for the future and the ‘new normal’, though evidence on student compliance and academic outcomes are yet to be explored.

Finally, it was encouraging to see that students’ understanding of nephrology following this speciality block of training was greater than for those prior to nephrology exposure. Indeed, this was a phenomenon not seen with certain specialities (including the ‘core’ medical subjects of cardiology, respiratory medicine and gastroenterology) and was only mirrored by rheumatology and geriatric medicine. This effect may well be a representation of the ‘baseline’ level of confidence and that there is a narrower scope for improvement in specialties that students initially feel confident with. Yet the caveats to this were that the actual levels of understanding remained lower in nephrology (and indeed in rheumatology and geriatrics) than in these other subjects, and also that no differences were seen for other domains in the survey when students pre- and post- nephrology exposure were compared. Thus, there remains work to be done.

But there are factors, aside from the delivery of teaching, which influence students’ career choices, and in regard to nephrology some interesting themes arise. The most highly rated area which was attractive to students was the ability to make a difference to patients, an overarching theme reflecting the altruistic nature of medical students [14]. It is encouraging that this is an attractive feature of the specialty, and we find it encouraging that the teamworking element of nephrology also featured high in the list of attractions. Work-life balance was also cited highly by the respondents to this study as an attractive feature of the specialty. This is an interesting perspective as the work-life balance of nephrology is not classically considered to be an attractive feature of the specialty amongst nephrologists themselves [4]. Although these views of the students may represent naivety or lack of first-hand exposure to real-life clinical practice, it is also conceivable that these historic (some might say entrenched) views are outdated. Certainly, the clinical nephrology departments have significant numbers of part-time senior (and junior) nephrologists, which one might consider a surrogate for, or at least an attempt towards, the work-life balance that is perceived by students.

At the other end of the spectrum, complexity of pathology, prestige of the speciality and the required length of training were cited as neither attractions of nor dissuaders towards nephrology. Certainly, the subject is indeed complex [3], and has been perceived as such in previous studies which addressed nephrology as a comparator speciality to neurology [5, 6]. As such, we can perhaps consider complexities of nephrology in the same light as those within neurology, making the case for ‘nephrophobia’. In light of this, it is perhaps understandable and maybe actually beneficial that students seemed to judge the prestige of the speciality in somewhat neutral terms. Neutrality was also displayed regarding length of training, which is the same as that for all core medical specialties [15], something that seems to be implicitly recognised by the students. Of course, there is no comparator here for us to discuss or explore the complexities of this finding, perhaps an area for further exploration.

The opportunities for research in nephrology are many and it is traditionally seen as a research-rich specialty [16]. Yet, this area was not scored especially highly amongst the respondents to this survey. It is possible this is a ‘centre-specific’ effect, and maybe the opportunities for renal research are not as visible as they should be; it may also represent changing approaches to traditional renal research, with an evolving focus on clinical and translational, rather than purely laboratory based and basic science research. This is an area that justifies greater exploration to understand the drivers to students’ responses.

Finally, it is logical to think that the impressions of nephrologists themselves influence the decision to choose one speciality over another, and so we asked this question specifically, broadly taking into account the ‘cognitive’ and ‘affective’ domains of students’ attitudes towards clinicians. Cognitive domains of intelligence, academic ability and hardworking scored high in students’ perceptions of nephrologists, with most agreeing or strongly agreeing with the operator statements. Although still positively referenced, the affective domains of caring, friendliness and approachability were more cautiously scored (although we accept that it ambiguous as to the ‘object’ of these affects – i.e. towards patients, or towards students, general or situated). So, whilst nephrologists are not seen as being unfriendly or unapproachable, the results lend credence to the notion that nephrology is predominantly seen as a highly academic specialty which requires high-level work ethic and intelligence.

We acknowledge that the results of this study ostensibly represent a ‘single-centre’ experience (from the perspective of the academic institution as described above), with students’ opinions coloured by unique experiences, but the results resonate with the inescapable observation of the waning ability for the speciality to attract the next generation of trainees [1, 2], and offer some explanation as to why that might be. In light of the diversity of clinical exposure and breadth of experience for the students in this study, we believe that generalisability and applicability beyond the current population is likely, and future examination across centres would be very welcome. The exploration of these issues is in its infancy, and we hope the current work informs further study and intervention.

In sum, the data presented here represent three distinct themes that may contribute to the impressions of nephrology among students. Firstly, that nephrology is considered among students to be a highly academic specialty, something which may be a negative driver for some when it comes to career choices. Secondly, there is a lack of confidence amongst students as to their comprehension of nephrology which drives a negative impression of the specialty as a potential career. Thirdly, many students appear to hold pre-conceptions of the specialty which are not entirely born-out in real nephrological practice, something which needs to be addressed with more real-world experience if the specialty is to attract more doctors to it at an early-career stage. We hope that in the same way that Flanagan et al. [5] inspired further investigation in the case of Zinchuk et al. [6], this may begin a further exploration on a national rather than regional or even international scale of the issues surrounding the future of the renal profession.

## Supplementary Information

**Additional file 1.** Survey Questions

## Data Availability

The datasets used and/or analysed during the current study are available from the corresponding author on reasonable request – Dr. William Hull.
