# Supplementary material for: Nephrophobia: a retrospective study of medical students’ attitudes towards nephrology education
Source: BMC Med Educ. 2022 Sep 9;22:667. doi: 10.1186/s12909-022-03713-z (PMC9461189; doi:10.1186/s12909-022-03713-z)
Supplement: Supplementary file 1 — Additional file 1. Survey Questions [file 12909_2022_3713_MOESM1_ESM.docx]

Survey Questions

Each question was repeated for each iteration e.g. 1a. I have a good knowledge of cardiology 1b. I have a good knowledge of respiratory.

1. I have a good knowledge of this subject…(Cardiology, Respiratory, Gastroenterology, Neurology, Rheumatology, Endocrinology, Geriatrics, Nephrology) Strongly agree/Agree/Neither agree nor disagree/Disagree/Strongly disagree
2. I would be confident in diagnosing a patient with this condition long term…(Cardiological, Respiratory, Gastroenterological, Neurological, Rheumatological, Endocrinological, Geriatric, Renal) Strongly agree/Agree/Neither agree nor disagree/Disagree/Strongly disagree
3. I would be confident in managing a patient with this condition in the acute setting…(Cardiological, Respiratory, Gastroenterological, Neurological, Rheumatological, Endocrinological, Geriatric, Renal) Strongly agree/Agree/Neither agree nor disagree/Disagree/Strongly disagree
4. I have a good understanding of the pathophysiology and aetiology of this condition…(Cardiological, Respiratory, Gastroenterological, Neurological, Rheumatological, Endocrinological, Geriatric, Renal) Strongly agree/Agree/Neither agree nor disagree/Disagree/Strongly disagree
5. I find this area of nephrology easy to comprehend…(Anatomy, Physiology, Interpreting investigations, Dialysis, Pharmacology, Surgery/transplantation, Interactions with other body systems) Strongly agree/Agree/Neither agree nor disagree/Disagree/Strongly disagree
6. I would feel more confident with nephrology if I had more teaching of this at medical school…(Anatomy, Physiology, Interpreting investigations, Dialysis, Pharmacology, Surgery/transplantation, Interactions with other body systems) Strongly agree/Agree/Neither agree nor disagree/Disagree/Strongly disagree
7. This is a good way to learn nephrology…(Bedside teaching, Attending lectures, Lectures, Attending ward rounds, Online resources/tutorials) Strongly agree/Agree/Neither agree nor disagree/Disagree/Strongly disagree
8. This factor of renal practice attracts me to the field…(Ability to make a difference, Work-life balance, Teamworking, Variety of pathology, Possibility to perform procedures, Research opportunities, Earning potential, Complexity of pathology, Prestige/respect, Length of training) Strongly agree/Agree/Neither agree nor disagree/Disagree/Strongly disagree
9. When I think of nephrologists, I consider them be...(Intelligent, hardworking, Academic, Friendly, Approachable, Caring) Strongly agree/Agree/Neither agree nor disagree/Disagree/Strongly disagree
10. Gender
11. Year of study (completed)
